# Matching geographical assignment by stable isotopes with African non-breeding sites of barn swallows *Hirundo rustica* tracked by geolocation

**DOI:** 10.1371/journal.pone.0202025

**Published:** 2018-09-14

**Authors:** Nina Seifert, Roberto Ambrosini, Luana Bontempo, Federica Camin, Felix Liechti, Diego Rubolini, Chiara Scandolara, Nicola Saino, Steffen Hahn

**Affiliations:** 1 Michael Succow Foundation for the Protection of Nature, Greifswald, Germany; 2 Department of Earth and Environmental Sciences (DISAT), University of Milano Bicocca, Milano, Italy; 3 Dipartimento Qualità Alimentare e Nutrizione, Fondazione E. Mach—Istituto Agrario di San Michele all'Adige, San Michele all'Adige, Italy; 4 Department of Bird Migration, Swiss Ornithological Institute, Sempach, Switzerland; 5 Dipartimento di Bioscienze, Univ. degli Studi di Milano, Milano, Italy; Fred Hutchinson Cancer Research Center, UNITED STATES

## Abstract

Knowledge on whereabouts within the annual cycle of migratory species is prerequisite for many aspects in ecology and biological conservation. Spatial assignments of stable isotopes archived in tissues allows for later inference on sites where the specific tissue had been grown. It has been rarely tested whether spatial assignments match directly tracked non-breeding residences, especially for migratory songbirds. We here compare assignments of stable isotopes from feathers of Palaearctic Barn swallows *Hirundo rustica* with their African non-breeding residence sites tracked by geolocation.Assignments based on δ^2^H, δ^13^C and δ^15^N isotope compositions delineate three main non-breeding regions: a main cluster in central Africa, a second in West Africa, and the third cluster in Northern Africa. Using δ^13^C, δ^15^N only, non-breeding sites ranged from clusters in West/Southwest Africa to South East Africa with a centre in Central Africa. The non-breeding areas (50% and 75% Kernel density estimates, KDE) of the birds tracked by geolocation stretched from West Africa via central Africa to southern Africa. We found little overlap of 0.3% (assuming a 1:1 odds ratio) to 1.4% (3:1 odds ratio) in the three element assignments and KDEs for only 2 and 13 individuals out of 32 birds. Assignment maps for two elements (δ^13^C, δ^15^N) and KDEs showed higher consistencies with an overlap of 3.6 and 8.5% for 12 and 18 birds. We argue that the low matching between stable isotope assignments and non-breeding sites in our study arise from insufficient baseline data for Africa (concerning both isoscapes and specific discrimination functions). However, other factors like aerial foraging habit of the species, and a potential mismatch of non-breeding site location and the spatial origin of aerial plankton might further hamper accurate assignments. Finally we call for concerted analyses of tissues i.e. feathers and claws of birds which are grown at known sites across the continent and from species with various ecological requirements (diverse habitats, foraging behaviours, and diet compositions) to establish isoscapes for general applicability.

## Introduction

Individual residence sites outside the breeding season are still fragmentary known for many populations of migratory animals. Underlying reasons are e.g. body size constraints which impede the adoption of transmitting devices such as GSM and ARGOS PTTs, low recapture rates of individuals marked with archival tags (GPS, geolocators) [1] or even the low numbers in rare species. This lack of knowledge is unsatisfactory as migratory species are assumed to be prone to divergent changes in various environments during their annual cycle [2] and thus their actual distribution should be identified urgently to be able to track ongoing and future distributional shifts [3].

During the last decade, indirect methods for the identification of the distribution in long-distance migrants such as small passerine birds or insects became progressively more sophisticated and nowadays enable an outline of the species’ whereabouts on a very fine geographical scale [4]. The accuracy of localisation attempts seems especially crucial when targeted conservation actions subsequently are planned within the identified areas.

The analysis of naturally occurring stable isotopes archived in animal tissues is one of the most widely adopted indirect tracking method developed to date [5]. Stable isotope analyses are very powerful tools to observe various ecological phenomena in animals, related to food and water intake, metabolism and finally the incorporation of chemical elements into the animal’s tissues. Herein, the tissue’s isotopic composition mirrors the source composition of the diet in a predictable manner [6]. Moreover, various tissues in a broader sense differentially archive this information on temporal scales from hours, like the composition of breath and blood plasma [7], to very long times in metabolically inert tissues like teeth [8], keratin in claws and feathers [9,10] or in hair of ancient mummies [11].

Additionally, the distribution of many stable isotopes like δ^13^C, δ^2^H and δ^18^O shows distinct spatial pattern across broad geographical scales, allowing for geographical assignments of archive stable isotope compositions and thus inference on animal [12]. Today this approach is frequently used especially in the study of long-distance migratory animals whose distribution during parts of the annual cycle remains unknown so far. Prominent examples include diverse animal classes like insects [13, 14], mammals [15] and most frequently birds [16].

The approach matches the isotopic composition of a tissue, whose time of synthesis is approximately known, with the geographically specific isotope composition of the diet ingested during the focal time [17, 18]. The method, however crucially depends on contrasting geographical differences in the composition of certain elements and on detailed and complete data for ground-truthed base line maps (so called isoscapes). Unfortunately, the latter usually exist mainly for northern hemisphere regions [19]. Thus, predictions with sufficiently high confidence are possible for certain regions on Earth, whereas for other areas like the African continent such predictions might be hard to establish [20].

Assignments can be more powerful, if several chemical elements with various geographical patterns are combined. In a pioneering work, [21] proposed a detailed method for geographical assignments of Afrotropical migrant birds based on three natural stable isotopes, namely ^2^H, ^13^C and ^15^N, wherein the baseline modelling is done by means of a multi-isotopic cluster model. This approach was refined, among others, by [4] by applying multivariate normal probability density functions for a spatially explicit assignment of the moult origin of birds. The method is based on a high assignment resolution (e.g. 0.33° [4]) instead of the rather broad attribution to one of four or five isotopic similar regions (clusters) delineated by [21]. However, despite its increasing application, very few studies tested the assignment accuracy by matching assignment results with parallel direct tracking data such as geolocation [22, 23, 24]. To our best knowledge, the isotopic assignment of moult origins in birds in the African continent has never been previously validated using individual-based migration tracking data.

We here compared the geographical assignment to certain regions in Africa by stable isotope ratios with non-breeding residence sites determined by geolocation of widespread Palearctic–Afrotropical migrant, the Barn swallow *Hirundo rustica*. Barn swallows migrate from their breeding sites in Europe to sub-Saharan non-breeding sites, which range from West Africa to South Africa depending on the specific population [25, 26, 27]. Thus, the species occupies regions with very different habitats and contrasting isotopic conditions [28]. Barn swallows usually arrive in the non-breeding grounds at the end of September/beginning of October, where they remain stationary for about six months until spring departure [29]. At their non-breeding sites, adult Barn swallows accomplish the moult of wing feathers [30]. Consequently feathers grown during the non-breeding period within Africa should reflect the stable isotope composition of prey and water available at these particular non-breeding sites and within a confined period (e.g. growing season). In our study, we tested the hypothesis that regions derived from spatial assignment of stable isotope composition of African-grown feathers match the African non-breeding sites derived by geolocator tracking.

## Material and methods

### Study system and geographical assignment by light-level geolocation

We used data from a geolocation study on the non-breeding distribution of individual Barn swallows breeding in southern Switzerland (about 46°N, 9°E) and northern Italy (about 45°N, 9°E) [29]. Birds had been equipped with geolocators (type SOI-GDL2, Swiss Ornithological Institute) in spring 2010 and 2011. After spring arrival in the subsequent year, geolocators were collected and a feather sample from the wing (the innermost tertial) of returning birds at their respective breeding sites (for details on the geolocator study see [31, 29]). Non-breeding sites were distributed in sub-Saharan Africa from Mali and Senegal in the West to South Africa; the main residences were located in the region of Cameroon to Nigeria where about 88% of the studied individuals overwintered in a 1000 km wide area [29].

**Fig 1:**
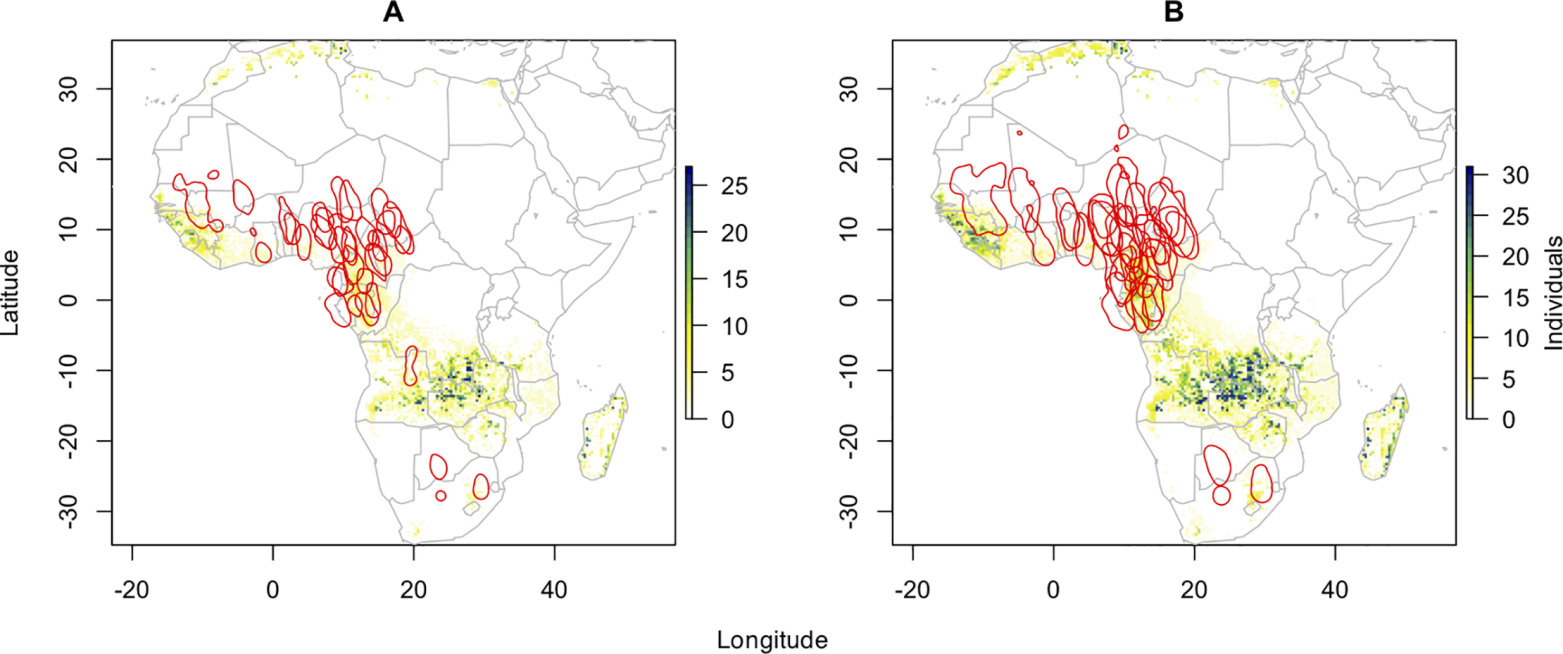
Predicted moult origin/nonbreeding regions for barn swallows based on isotope assignment for three isotopes δ^2^H, δ^13^C and δ^15^N and geolocation. Kernel density estimates are shown in red. The colours indicate the number of individuals that were isotopically consistent with a given raster cell in the isoscape representing the likely moulting site. a) Odd ratio 1:1 and 50% KDE. b) Odd ratio 3:1 and 75% KDE.

For the matching of isotope assignments and geolocation, we used a subset of 32 birds (22 males, 10 females, from 2010/11 and 4 from 2011/12), which represent the entire non-breeding range with three individuals each for West and southern Africa) as well as a random selection of 26 birds for the main nonbreeding grounds for this studied population (see above and Fig 1). The individual non-breeding areas were determined by 50% and 75% kernel density estimations (KDE) (300 km search radius, ArcGIS 9.3) using geolocator location during the nonbreeding period, i.e. after arrival in the beginning of October and before departure in the beginning of March from sub-Saharan nonbreeding sites. Wing moult in barn swallows is usually performed entirely during the non-breeding period [30], thus we assume the wing feather had been completely grown at these non-breeding sites.

### Stable isotope analysis

The feather samples were cleaned with hexane to remove contaminations, and subsequently air-dried under a fume hood in the laboratory. For δ^13^C and δ^15^N determination, about 0.3 mg of the feather vane samples were weighed into tin capsules. Their determination in one run was carried out using an isotope ratio mass spectrometer (Isoprime, Elementar Analysensysteme GmbH, Germany) interfaced with an elemental analyser (Vario Isotope Cube, Elementar Analysensysteme GmbH, Germany). For the analysis of δ^2^H, about 0.2 mg of sample was placed in silver capsules and, once weighed, the samples and reference materials were left in laboratory air moisture for at least 96 h, then placed in a desiccator with P_2_O_5_ under vacuum for a further 96 h. Samples were then loaded onto the autosampler tray, put on the carousel, sealed with a cover and purged with argon. δ^2^H was determined using an isotope ratio mass spectrometer equipped with a TC/EA (thermo combustion pyrolyser—elemental analyser; Delta Plus XP -ThermoFinnigan, Bremen, Germany).

The isotope ratios were expressed in δ against V-PDB (Vienna—Pee Dee Belemnite) for δ^13^C, Air for δ^15^N and V-SMOW (Vienna—Standard Mean Ocean Water) for δ^2^H according to [32]. The values of δ^2^H were calculated building a regression line through the two reference materials Caribou Hoof Standard (CHS, USGS—United States Geological Survey, Reston Stable Isotope Laboratory, Virginia, USA) and Kudu Horn Standard (KHS, USGS). The reference values of the reference materials considered were δ^2^H = -197.0 ‰ for CBS and δ^2^H = -54.1 ‰ for KHS. Therefore, the δ^2^H values of the samples were expressed in comparison to V-SMOW on scales normalized in such a way that the δ^2^H value of SLAP (Standard Light Antarctic Precipitation) was -428 ‰, as recommended by IUPAC [33]. In each analytical sequence, analysis of an internal quality control material (keratin, Camida Ltd., Tipperary, Ireland) was included to check analytical system performance.

δ^13^C and δ^15^N isotopic values were calculated against in-house standards, which were themselves calibrated against international reference materials: fuel oil NBS-22 (IAEA International Atomic Energy Agency, Vienna, Austria; -30.031 ‰) and sugar IAEA-CH-6 (-10.449 ‰) for δ^13^C, L-glutamic acid USGS 40 (-26.389 ‰ and -4.5 ‰ for δ^13^C and δ^15^N), hair USGS 42 (δ^15^N = +8.05 ‰ and δ^13^C = -21.09 ‰) and USGS 43 (δ^15^N = +8.44 ‰ and δ^13^C = -21.28 ‰) for ^13^C/^12^C and ^15^N/^14^N.

Method uncertainty (calculated as one standard deviation in repeatability conditions) was 0.1 ‰ for δ^13^C, 0.2 ‰ for δ^15^N and 2 ‰ for δ^2^H.

### Geographic assignment to the moult origin by stable isotopes

We aimed at identifying the moulting area during the nonbreeding period by applying a multiple element approach [21, 4]. We used feather isotope data of δ^13^C, δ^15^N and δ^2^H with a resolution 0.33° to perform spatially explicit assignments to isoscapes of Africa. The feather isoscapes were derived from 1) isoscapes of amount-weighted mean growing season δ^2^H in precipitation (δ^2^H_p_, [34]), 2) the theoretical spatial δ^13^C distribution of plants [35] and 3) plant δ^15^N isoscape developed by [36], following the method developed by [21]. Thereby, the δ^2^H_p_ isoscape (p-precipitation) was converted into a δ^2^H_f_ isoscape (f-feather) based on regression parameters derived from a regression of δ^2^H_f_ in feathers of insectivorous Eurasian reed warblers (*Acrocephalus scirpaceus*) against δ^2^H_p_ [37]. Discrimination between plant and feather δ^13^C and δ^15^N isoscapes were accounted for by a discrimination factor of +2‰ for δ^13^C and +5‰ for δ^15^N [21].

We determined the likelihood that a given raster cell within the feather isoscapes represents a potential moult origin of a particular feather by applying multivariate normal probability density functions (mvnpdf) following [4]. Thereby, we firstly considered the set of three isotopes δ^13^C, δ^15^N and δ^2^H, and then we repeated the procedure based only on the set of two isotopes δ^13^C and δ^15^N resulting in two different probability surfaces for every individual. We did not include the species’ known nonbreeding distribution as prior information in the assignment procedure.

In a second step, we reclassified each raster cell into likely (1) and unlikely (0) by selecting those geographic locations that fell within the upper 50% (1:1 odd ratio) and 75% (3:1 odd ratio) of the spatially explicit probability densities within the likelihood map. These values were chosen as they correspond to the 50% and 75% KDE of the geolocator locations. The resulting individual binary assignment maps were summed up to depict the likely population moult origin.

Furthermore, in order to test the applicability of the isotopic clusters proposed by [21] we estimated the assignment of individual feather stable isotope (SI) values to four and five isotopic clusters (identified by [21]) derived by cluster analysis based on δ^13^C, δ^15^N and δ^2^H or δ^13^C and δ^15^N, respectively. In this assignment procedure, linear discriminant function analysis (DFA) is used to predict the posterior probability that a sample with a given multi-isotope composition could have originated from any given cluster within Africa, given the predicted ranges (min, max, mean and SD) for feather δ^13^C, δ^15^N and δ^2^H [21].

All assignments were performed by using the “mvnmle”, “raster” and “maptools” packages [38, 39, 40] in R 3.1 [41].

### Matching geographical assignment by stable isotopes and geolocation

We quantified the overlap of isotopic assignment and geolocation by calculating the percentage of raster cells of likely moult origin (by stable isotopes) overlapping with the individual 50% and 75% KDE derived from geolocation.

In addition, we calculated the percentage of cells within 50% and 75% KDE assigned to the four (δ^13^C, δ^15^N and δ^2^H) and five (δ^13^C, δ^15^N) isotopic clusters based on the cluster analysis by [21].

This study was carried out under permission #301 of Progetto Rondine Sul Piano di Magadino, Italy. All efforts were made to minimize handling time of the birds while geolocators were attached and removed.

## Results

### Identification of nonbreeding areas by geolocation

The non-breeding areas of the subset of Barn swallows used in this study stretched from 5°W to 31°E longitude and 15°N to 28°S latitude (Fig 1, for the complete data set see [29]) including a variety of main vegetation types from grass savannah (West Africa), tree savannah and tropical forest (eastern west and central Africa) to mainly steppe habitats (southern Africa). The median area of non-breeding sites calculated as kernel density area comprised about 149,020 km^2^ (25–75%: 111,495–171,191 km^2^) for the 50% KDE and 345,730 km^2^ (25–75%: 255,200–418,764 km^2^) for the 75% KDE. This corresponds to approximately 107 and 252 cells for the stable isotope assignment (see below).

### Geographical assignment by stable isotopes

Assignment based on stable isotope composition of three elements, i.e. δ^2^H, δ^13^C and δ^15^N highlighted four main regions comprising the likely moult origins (Fig 1A and 1B). For 31 of 32 individuals, origins were located in raster cells south of 5°S, in DR Congo, Zambia, Angola and neighbouring countries as well as Madagascar. Furthermore, a third cluster of likely moult origin was found in West Africa (Guinea Bissau—Liberia), while a third cluster comprised cells close to the coastlines in Northern Africa (Morocco, Tunisia and the Nile delta in Egypt). On average a likely moult origin was assigned for 414 cells (1:1 odds) and for 953 cells (3:1 odds; Table 1).

**Table 1:** Overview of geographical position (Latitude and Longitude), size of nonbreeding range of Barn swallows (KDE-Kernel density estimates in km^2^ and number of raster cells), the number of assigned raster cells based on stable isotope assignments (CN = two isotopes: δ^2^H, δ^13^C and HCN = three isotopes δ^2^H, δ^13^C and δ^15^N) and overlap of matching raster cells (KDE & SI assignment, the number of cells and %).

ID	Lat (°)	Long (°)	Size KDE (km^2^)		Size KDE (# cells)		Assignment based on CN (# cells)		Assignment based on HCN (# cells)		Overlap KDE and assignments HCN (# of cells, %)				Overlap KDE and assignments CN (# of cells, %)			
			50 %	75 %	50 %	75 %	odds 1:1	odds 3:1	odds 1:1	odds 3:1	50 %	75 %	50 %	75 %	50 %	75 %	50 %	75 %
1RZ	4.0	14.6	171826	348808	133	269	828	1420	312	813	0.0	3.0	0.0	1.1	6.0	66.0	4.5	24.5
1ST	9.2	-2.9	127633	281490	98	217	1671	3143	770	1578	0.0	1.0	0.0	0.5	0.0	11.0	0.0	5.1
1SU	-0.4	14.2	133563	268400	103	207	866	1824	400	1040	0.0	21.0	0.0	10.1	7.0	71.0	6.8	34.3
1TQ	13.5	10.3	183776	444172	142	343	1073	1771	415	1006	0.0	0.0	0.0	0.0	0.0	0.0	0.0	0.0
1TS	12.6	-8.0	124394	324231	96	250	341	1093	202	726	0.0	0.0	0.0	0.0	0.0	0.0	0.0	0.0
1UE	9.5	7.4	91977	201018	71	155	920	1556	405	1011	0.0	0.0	0.0	0.0	0.0	0.0	0.0	0.0
1UH	8.6	6.6	139026	329324	107	254	1169	2004	194	462	0.0	0.0	0.0	0.0	0.0	0.0	0.0	0.0
1UJ	10.2	16.0	269300	522239	208	403	1147	1931	560	1241	0.0	0.0	0.0	0.0	0.0	0.0	0.0	0.0
1UY	10.1	17.1	278742	551148	215	425	1021	1688	584	1160	0.0	0.0	0.0	0.0	0.0	0.0	0.0	0.0
1WG	6.4	10.5	155518	322219	120	249	914	1541	199	464	0.0	0.0	0.0	0.0	0.0	5.0	0.0	2.0
1WH	0.9	13.0	102288	297575	79	230	1636	3434	620	1289	0.0	0.0	0.0	0.0	0.0	21.0	0.0	9.1
1WW	1.5	8.7	139341	458723	108	354	1031	1789	175	414	0.0	0.0	0.0	0.0	17.0	84.0	15.8	23.7
1XR	7.0	10.6	115981	323817	89	250	866	1466	197	515	0.0	0.0	0.0	0.0	0.0	0.0	0.0	0.0
1XT	3.6	11.5	171685	344246	132	266	845	1494	225	626	0.0	3.0	0.0	1.1	14.0	63.0	10.6	23.7
1YA	-1.3	8.9	173642	420043	134	324	1295	2526	647	1363	0.0	12.0	0.0	3.7	18.0	78.0	13.4	24.1
1YD	-27.9	25.6	125560	233820	97	180	1401	2684	929	1899	7.0	2.0	7.2	1.1	4.0	1.0	4.1	0.6
1YW	7.7	7.3	202535	414928	156	320	166	855	113	507	0.0	0.0	0.0	0.0	0.0	0.0	0.0	0.0
1ZS	0.6	6.3	101523	242870	78	187	996	1710	227	570	0.0	0.0	0.0	0.0	8.0	37.0	10.2	19.7
1ZV	3.3	15.6	160011	307053	123	237	790	1397	267	748	0.0	0.0	0.0	0.0	3.0	27.0	2.4	11.4
2AA	12.7	6.2	157142	329983	121	255	1114	1934	110	282	0.0	0.0	0.0	0.0	0.0	0.0	0.0	0.0
2AI	13.1	-10.4	294337	631533	227	487	592	1349	201	520	0.0	11.0	0.0	2.3	0.0	0.0	0.0	0.0
2AL	7.6	13.2	147322	456900	114	353	1177	2001	470	1055	0.0	3.0	0.0	0.9	0.0	1.0	0.0	0.3
2AR	6.3	9.1	198118	404295	153	312	134	888	148	592	0.0	0.0	0.0	0.0	0.0	1.0	0.0	0.3
2AZ	9.1	15.9	111478	212401	86	164	1253	2321	643	1377	0.0	0.0	0.0	0.0	0.0	0.0	0.0	0.0
2BJ	7.4	-0.3	111544	220143	86	170	968	1662	225	476	0.0	0.0	0.0	0.0	0.0	0.0	0.0	0.0
2CT	5.4	7.8	124339	323513	96	250	909	1537	407	963	2.0	16.0	2.1	6.4	1.0	6.0	1.0	2.4
2DC	-3.6	14.0	149296	340756	115	263	1042	2327	523	1194	0.0	17.0	0.0	6.5	16.0	92.0	13.9	35.0
2DF	7.2	18.4	156047	498641	120	385	1189	2082	138	336	0.0	0.0	0.0	0.0	0.0	1.0	0.0	0.3
3CX	4.9	-4.5	60807	210245	47	162	767	1374	464	975	0.0	1.0	0.0	0.6	0.0	0.0	0.0	0.0
3RD	-2.8	14.5	95104	343880	73	265	988	2200	437	1043	0.0	14.0	0.0	5.3	20.0	114.0	27.3	43.0
3RN	-25.0	20.6	105973	250801	82	194	1567	3235	1142	2412	0.0	0.0	0.0	0.0	0.0	0.0	0.0	0.0
3ST	7.9	-2.7	96932	204126	75	158	1877	3448	915	1841	0.0	9.0	0.0	5.7	5.0	20.0	6.7	12.7
**mean**			**149274**	**345729**	**115**	**267**	**1017**	**1928**	**415**	**953**	**0.3**	**3.5**	**0.3**	**1.4**	**3.7**	**21.8**	**3.6**	**8.5**

**Fig 2:**
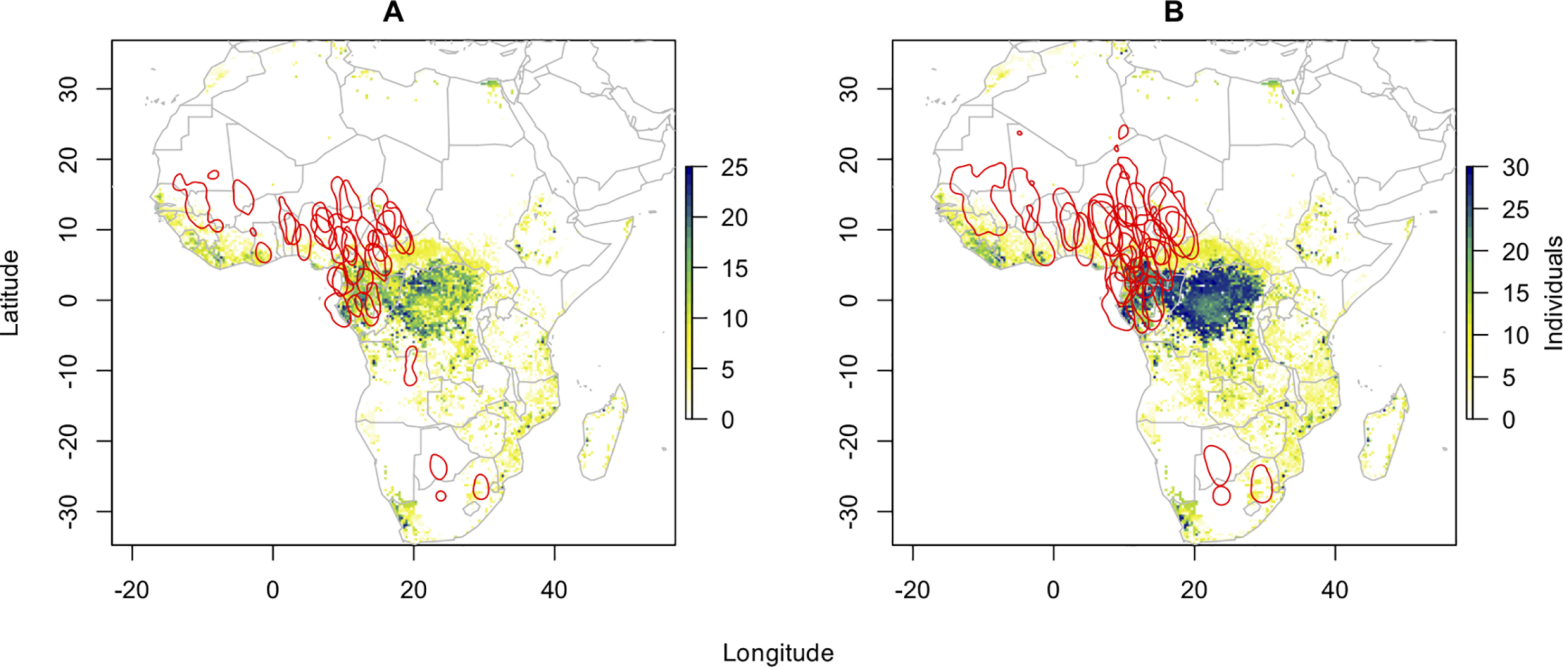
Predicted moult range/nonbreeding range for barn swallows based on isotope assignment for two isotopes δ^13^C and δ^15^N and geolocation. The different colours of raster cells indicate the number of individuals that were isotopically associated with a given raster cell of the isoscape. Kernel density estimates (KDE) derived from geolocation are shown in red. a) Odd ratio 1:1 and 50% KDE. b) Odd ratio 3:1 and 75% KDE.

Assignments derived by two stable isotopes, i.e. δ^13^C and δ^15^N, were less distinct with a mean number of 1017 assigned cells for the 1:1 odds and 1928 assigned cells for 3:1 odds (Table 1). Herein, likely moult origins ranged from a cluster in sub-Saharan West Africa to South West and South East Africa with a clear agglomeration of individual assignments for Central Africa (Cameroon, DR Congo, Central African Republic). A small cluster again was located in the Nile delta (Fig 2A and 2B).

The discriminant function analysis predominantly assigned feather isotope values to isotopic cluster 1 and 2 based on three isotopes δ^2^H, δ^13^C and δ^15^N with an average likelihood of 0.89 (Table 2). Considering only δ^13^C and δ^15^N, the bulk of individuals was clearly assigned to cluster 3 with only five individuals assigned to cluster 2. Average likelihood amounted to 0.99 (Table 3).

**Table 2:** Assignment of individual feather stable isotope value to four isotopic clusters based on δ^2^H, δ^13^C and δ^15^N (according to [21]) and the percentage of raster cells assigned to four isotopic clusters within the individual 50% KDE and 75% KDE. Grey shading indicates corresponding assignment of individual feather stable isotopes and KDE.

	Assignment Isotopes				Cells within 50% KDE				Cells within 75% KDE			
SampleID	C1	C2	C3	C4	C1	C2	C3	C4	C1	C2	C3	C4
1RZ	100	0	0	0	23	76	1	0	41	52	8	0
1ST	0	100	0	0	0	0	100	0	0	0	98	2
1SU	4	96	0	0	73	27	0	0	76	24	0	0
1TQ	88	12	0	0	0	0	61	39	0	0	65	35
1TS	98	2	0	0	0	0	100	0	0	0	100	0
1UE	100	0	0	0	0	0	100	0	0	2	98	0
1UH	40	60	0	0	0	6	94	0	0	6	94	0
1UJ	78	22	0	0	5	61	34	0	4	57	39	0
1UY	100	0	0	0	0	0	81	19	4	73	23	0
1WG	100	0	0	0	9	32	60	0	10	29	62	0
1WH	0	100	0	0	97	3	0	0	73	26	1	0
1WW	32	68	0	0	91	9	0	0	57	21	21	0
1XR	100	0	0	0	2	48	51	0	1	28	72	0
1XT	86	14	0	0	57	39	4	0	54	28	18	0
1YA	0	100	0	0	89	5	5	0	89	8	3	0
1YD	0	100	0	0	41	58	1	0	29	64	7	0
1YW	100	0	0	0	0	4	96	0	0	5	95	0
1ZS	52	48	0	0	88	12	0	0	79	21	0	0
1ZV	100	0	0	0	15	83	2	0	22	61	17	0
2AA	34	56	0	0	0	0	79	21	0	0	73	27
2AI	56	44	0	0	0	22	78	0	0	23	75	2
2AL	12	88	0	0	0	13	87	0	0	15	68	17
2AR	88	12	0	0	12	18	69	0	17	15	68	0
2AZ	12	88	0	0	0	0	93	7	0	0	84	16
2BJ	100	0	0	0	1	4	94	0	2	4	94	0
2CT	100	0	0	0	15	34	51	0	12	27	62	0
2DC	0	100	0	0	73	27	0	0	76	23	1	0
2DF	2	98	0	0	1	1	98	0	16	13	70	1
3CX	100	0	0	0	20	2	78	0	9	1	89	1
3RD	0	100	0	0	74	26	0	0	68	21	11	0
3RN	0	100	0	0	0	0	100	0	0	0	100	0
3ST	0	100	0	0	0	0	100	0	0	0	100	0

**Table 3:** a) Assignment of individual feather SI value to five isotopic clusters based on δ13C and δ15N (according to [21]) and percentage of raster cells assigned to four isotopic clusters within individual 50% and 75% KDE. Grey shading indicates corresponding assignment of individual feather stable isotopes and KDE.

	Assignment Isotopes					Cells within 50% KDE					Cells within 75% KDE				
SampleID	C1	C2	C3	C4	C5	C1	C2	C3	C4	C5	C1	C2	C3	C4	C5
1RZ	0	0	100	0	0	0	78	22	0	0	0	58	39	4	0
1ST	0	100	0	0	0	0	0	0	59	41	0	0	0	51	49
1SU	0	0	100	0	0	0	30	70	0	0	0	26	74	0	0
1TQ	0	0	100	0	0	0	0	0	12	88	0	0	0	21	79
1TS	0	0	100	0	0	0	0	0	1	99	0	0	0	24	76
1UE	0	0	100	0	0	0	3	0	97	0	0	3	0	79	18
1UH	0	0	100	0	0	0	6	0	94	0	0	7	0	83	10
1UJ	0	0	100	0	0	0	0	5	31	63	0	0	4	33	63
1UY	0	0	100	0	0	0	11	0	53	36	0	15	1	47	37
1WG	0	0	100	0	0	0	39	7	53	0	1	32	10	58	0
1WH	0	100	0	0	0	0	2	98	0	0	0	25	75	0	0
1WW	0	0	100	0	0	3	14	83	0	0	5	30	50	14	0
1XR	0	0	100	0	0	0	63	0	37	0	0	36	0	44	21
1XT	0	0	100	0	0	1	37	60	2	0	3	29	53	15	0
1YA	0	18	82	0	0	0	21	79	0	0	1	15	84	0	0
1YD	0	100	0	0	0	38	59	3	0	0	63	33	3	1	0
1YW	0	0	100	0	0	0	4	0	85	10	0	7	0	72	21
1ZS	0	0	100	0	0	8	20	72	0	0	12	24	63	0	0
1ZV	0	0	100	0	0	0	90	10	0	0	0	77	18	5	0
2AA	0	0	100	0	0	0	0	0	21	79	0	0	0	26	74
2AI	0	0	100	0	0	0	16	0	36	47	0	20	0	32	48
2AL	0	0	100	0	0	0	17	0	77	6	0	18	0	41	40
2AR	0	0	100	0	0	4	20	9	67	0	4	20	11	59	6
2AZ	0	0	100	0	0	0	0	0	45	55	0	0	0	45	55
2BJ	2	0	98	0	0	0	30	0	70	0	0	21	2	73	4
2CT	0	0	100	0	0	0	41	15	44	0	1	30	12	48	9
2DC	0	18	82	0	0	0	30	70	0	0	0	25	75	0	0
2DF	0	0	100	0	0	0	26	0	73	1	0	31	0	55	13
3CX	0	0	100	0	0	0	38	14	48	0	0	17	8	69	6
3RD	0	0	100	0	0	0	26	74	0	0	0	29	66	5	0
3RN	0	100	0	0	0	0	0	0	100	0	0	0	0	100	0
3ST	0	100	0	0	0	0	2	0	92	6	0	1	0	69	30

### Matching stable isotope assignment with geolocation

We found little overlap of stable isotope assignments based on δ^2^H, δ^13^C and δ^15^N and nonbreeding areas calculated as by KDE. Overlap increased when considering larger nonbreeding areas: For the 1:1 odds corresponding to the 50% KDE we found a match for only 2 individuals, whereas for the 3:1 odds and the 75% KDE 13 individuals (40%) showed some overlap (Table 1, S1 Fig).

However, the overlap between KDE and assigned cells was on average 0.3% for the 1:1 odds and 1.4% for the 3:1 odds (Table 1).

The assignments based on two isotopes (δ^13^C and δ^15^N), individual KDEs and isotope assignment maps were more consistent: 12 and 18 KDE comprised likely raster cells with a mean overlap of 3.6 and 8.5% (Table 1).

Similar to the little overlap between KDEs and isotope assignments based on 0.33° cells, there was hardly any consistency in the individual assignment to isotopic clusters and the cluster(s) represented by the cells inside the KDEs (Table 2). For the three-element cluster approach, only for three (50% KDEs) and two (75% KDEs) individuals, the assigned isotopic cluster corresponded with the cluster categories of cells inside the KDEs (with > 50% of raster cells representing one cluster; Table 2). For the two-element cluster approach (δ^13^C and δ^15^N) we found more matches between cluster assignments and cells within the KDEs: for eight (50% KDE) and seven individuals (75% KDE) clusters were the same for both methods (Table 3).

## Discussion

Our study highlighted a lack of concordance between the individuals’ assignment based on δ^2^H, δ^13^C and δ^15^N isotopes and the corresponding non-breeding sites derived from geolocation for Barn swallows during their non-breeding period in Africa.

This mismatch could be ascribed to several ecological as well methodological reasons. The expectation on a similarity of SI assignment and geolocation is based on some fundamental assumptions: (1) the geolocation kernel density estimate represents the bird‘s non-breeding residence site (during boreal winter), (2) the study species moult their wing feathers in their African non-breeding grounds. Further, (3), the diet ingested by swallows originates from the specific non-breeding site tracked by geolocators and finally (4) the isotopic composition of local diet is adequately reflected by the isoscape model. Each of these assumptions will be discussed below.

It is generally accepted that geolocation by light correctly determines locations based on predictive differences in sun rise and sun set times. However, the method has inherent inaccuracies caused by environmental shading and the behaviour of the tagged individual during sun rise/sun set times [42]. Generally, estimates of latitude are more inaccurate than longitude estimates, and inaccuracy is largest during equinox periods [43, 44]. In our study, we determined residence sites of Barn swallows during the boreal winter, and thus uncertainties due to equinox can be excluded. Moreover we used a rather conservative approach (KDE) for determining non-breeding areas, which may even most likely overestimate a Barn swallow’s home range during the non-breeding period. Thus, we would expect an overestimation in the overlap between non-breeding sites and SI assignment per individual instead of the general low percentage of matched raster cells per individual found in this study.

Correct spatial assignments based on stable isotope composition of e.g. feather keratin require prior knowledge about the time of formation of the targeted tissue. Barn swallows which moult outside the non-breeding season, e.g. at the end of the breeding season or *en route* during migration will certainly cause faulty assignments. Although there are some observations of wing moult had been already started on the European breeding grounds [45], the proportion of these early moulting birds in a population is generally very low with about 3% for Central European breeders [45]. Applying this percentage to our study, we would expect that not more than a single bird would carry a non-African isotope signature in its tertial wing feathers. Moreover, we can exclude the possibility that we simply could have missed the birds’ moulting period and thus site, as wing feather moult usually extents over almost the entire season when birds occur in sub-Saharan Africa [46]. Even acknowledging a small proportion of birds to have moulted their tertials outside the African non-breeding distribution, we would expect that isotopic composition in feathers would allow at least for some correct assignments.

Barn swallows are aerial feeders, preying on flying insects which might disperse over larger distances by wind drift. As dispersal distances of small insects like mosquitoes and blackflies can reach several hundreds of km when transported passively by winds [47, 48], influxes of alien isotope compositions could be a likely reason which has led to the mismatch of our geolocation and SI assignment results.

However, the prevailing wind directions in SW-Africa during the non-breeding season are rather NE or SW [49], which does not support a passive transport of insects from the Congo Basin (as suggested by SI assignments) to the majority of the Barn swallow’s non-breeding sites in Cameroon and Nigeria. It cannot be entirely excluded that the diet ingested by swallows originates, to some part, from outside the specific non-breeding site. However, the lack of knowledge both in diet selection during the non-breeding period as well as the spatial extent of both passive and active movements in prey insects within sub-Saharan Africa renders a conclusion impossible.

Consequently, we speculate we can exclude a significant effect of 1) potential inaccuracy of geolocation and 2) uncertainties in the timing and location of moult and as well do not consider 3) large-scale relocation of prey as a (major) probable cause for the mismatch found in our data set.

Rather, we assume that the reason for the discrepancy between isotopic assignment and actual residence of the birds is an inadequate reflection of isotopic composition, e.g. of local diet by the available isoscape models. Firstly, inconsistencies can be attributed to a mere lack of a sufficient data base, as the accuracy of modelled isoscapes naturally highly depends on underlying data. For the African continent, base data are less comprehensive than for other region on earth. For instance, the fundamental δ^2^H isoscape for Africa reflecting amount-weighted mean growing season δ^2^H in precipitation (δ^2^H_P_, [34]) is based on data from only 44 sampling stations which are not evenly distributed across the continent and for which years of collection also vary substantially [50].

Large inner- and inter-annual variation in δ^2^H_P_ caused by high fluctuations in the amount of precipitation [51, 52], and strong effects of local habitat due to evaporation [53] are further factors of uncertainty leading to an isoscape which might be too coarse for a precise delineation of individual distribution. In our data set, the limits of the available δ^2^H isoscape are already reflected in a relative improvement of assignment maps when we considered only δ^13^C and δ^15^N in the multivariate normal probability density functions. The overlap between KDE and isotope assignment clearly increased. However, the core areas of KDEs around 05°N and 12°E were still spatially very distant from those target regions based on δ^13^C and δ^15^N composition (core area approx. 01°S, 24°E; see further below).

Secondly, the low matching of stable isotope and geolocation data can be a matter of scale if the resolution of the available isoscapes and the size of the individual residence site deviate substantially. Although daily ranges of Barn swallows in Africa are still not known, there is indication that the species forages on a rather large scale as ring recoveries of the species within the same season encompassed distances of about 100 km. Furthermore, wide-ranging movements up to 600 km are recorded as well [54]. Accordingly, due to their potential wide-ranging aerial foraging behaviour, we would expect Barn swallows to integrate various isotopic values across the landscape much better than an e.g. rather sedentary species such as the Aquatic warbler (*Acrocephalus paludicola*), for which [55] recognized isotopic signatures being inappropriate markers for geographic assignments. Aquatic warblers are habitat specialists with very confined non-breeding home ranges of only a few hectares [56] the actual isotope value of a bird`s feather was interpreted to be largely determined by very small-scale foraging behaviour at a specific location rather than by large-scale isotopic gradients.

However, although the overall spatial scale reflected in the isotopic values of the individual Barn Swallows feathers should correspond to the resolution of the available isoscapes, there might still be effects of an uneven integration of isotopic composition (by the individual bird) across the landscape. This becomes especially evident when we excluded δ^2^H in the isotopic assignment due to uncertainties mentioned earlier. Based only on δ^13^C and δ^15^N, isotopic assignments of the majority of birds pointed to regions in the Congo basin, dominated by Guineo-Congolian evergreen and semi-deciduous rainforest [57]—and still hundreds of kilometres distant to the individual residence sites identified by geolocation.

Although the species’ ecology during the non-breeding season is fragmentarily known, authors assume Barn Swallows to primarily prefer riverbeds and wetland habitats for roosting and foraging [45, 58]. Azonal and local habitat types such as wetlands are known to harbour proportionally more C3 plants compared to the overall C3/C4 plant ratio in the predominating ecosystems [55]. This reveals isotopic values in local plants and herbivorous insects which are more similar to tropical forests than the grassland or savannah biomes identified by geolocation. Accordingly, despite the species’ rather large-scale foraging behaviour, feather isotope values could still deviate substantially from the prevailing δ^13^C gradients as isoscapes do not sufficiently account for such habitat effects on spatial scales below the landscape-level.

### Conclusion and outlook

The analysis of multiple stable isotopes for spatial assignments is a very powerful tool to derive geographical information. The method has been successfully applied to many terrestrial species and regions world wide (Europe/Asia: [59], North America: [60], South America: [61] and is very efficient to track individuals or carry-over effects in large sample sizes allowing for robust conclusions (i.e. [62]). Based on our dataset of 32 barn swallows we could not ground-truth isotopic assignments in Africa with areas localised by direct tracking using geolocation by light. We argue that this discrepancy may mainly arise from quality of isoscapes currently available for Africa and not from a general failure. As the evolution of sophisticated statistical methods at present theoretically enables prediction of probable moult origins with a resolution of 0.33° or higher, we caution against their precipitous use as long as underlying models for isoscapes based on a weak data basis, probably reflecting a much coarser resolution. We explicitly do not object to the application of isotopic assignments for Africa in general. As the method is low-cost, both in the lab and in the field, it is well suited for surveys accepting a (at present) coarse resolution [63, 27]. For studies requiring a finer resolution, the present base data for African feather isoscape(s) needs to be improved. Recently, there are endeavours to amend δ^2^H isoscape(s) by e.g. the application of feather samples with spatially explicit information [64]. However, beside the use of archival samples, systematic sampling and isotopic measurements of feathers of known origin and across several years are still fundamental for the delineation of a sound feather δ^2^H isoscape for Africa. Accordingly, tracking studies on species with moult in Africa should be accompanied by sampling of feathers allowing for further information on the isotopic conditions in the inferred locations.

So far, almost all assignment attempts have to be based upon African δ^2^H isoscapes which were calibrated by a function established for Eurasian reed warblers (*Acrocephalus scirpaceus*) sampled in Europe [37]. The development of a calibration function explicitly based on feathers of known origin grown within Africa might improve existing δ^2^H isoscapes significantly. However, these fine-tuning possibilities still have to be attended by a general improvement of the resolution and spatial arrangement of the δ^2^Hp-sampling stations (Global Network of Isotopes in Precipitation, GNIP) across the African continent [65].

Recently, there are large efforts to improve the existing GNIP data within the continent, i.e. IAEA is mapping surface water isotope values which can results in updated isoscapes which will likely be useful for marsh associated species or birds feeding on aquatic emergent insects [66].

Finally, we would like to call for a concerted action to solve the δ^2^H isoscape for Africa within the near future including sampling of species with different ecological requirements.

## Supporting information

S1 Fig Predicted moult origin/overwintering regions for two individual barn swallows based on isotope assignment for three isotopes δ^2^H, δ^13^C and δ^15^N and geolocation. 75% Kernel density estimates are shown in red. a) Example of best geographical overlap. b) Example of least overlap.
(TIFF)
