# Supplementary figures and images for: Matching geographical assignment by stable isotopes with African non-breeding sites of barn swallows Hirundo rustica tracked by geolocation
Source: PLoS One. 2018 Sep 14;13(9):e0202025. doi: 10.1371/journal.pone.0202025 (PMC6138371; doi:10.1371/journal.pone.0202025)

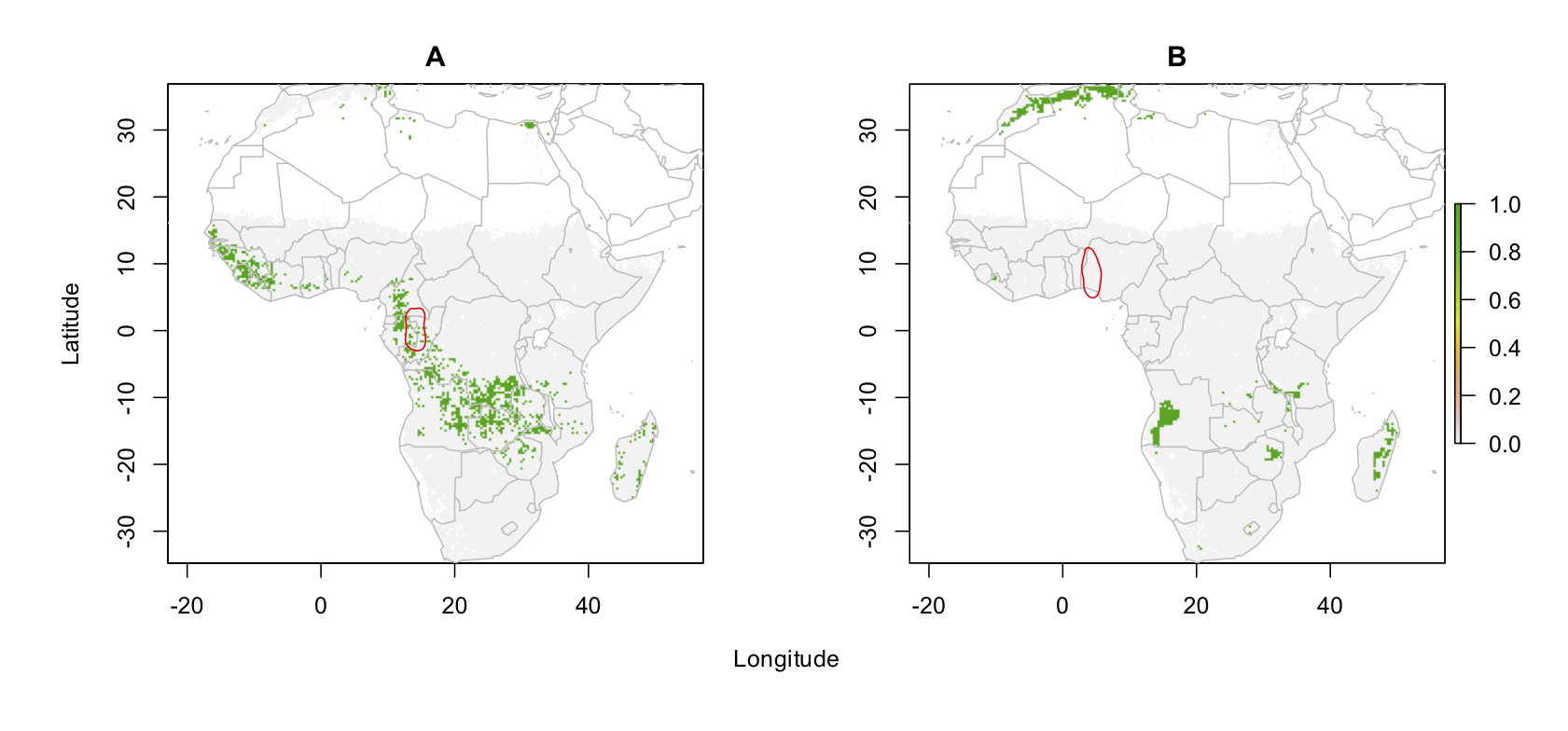

Supplement: S1 Fig — 75% Kernel density estimates are shown in red. a) Example of best geographical overlap. b) Example of least overlap. (TIFF) [file pone.0202025.s001.tiff]
